# Editorial: Migrants' psychosocial health: cultural and religious resources through resilience and coping

**DOI:** 10.3389/fsoc.2023.1286892

**Published:** 2023-10-03

**Authors:** Önver Andreas Cetrez, Selcan Kaynak

**Affiliations:** ^1^Faculty of Theology, Psychology of Religion, Uppsala University, Uppsala, Sweden; ^2^Department of Political Science and International Relations, Koç University, Istanbul, Türkiye

**Keywords:** resilience, coping, psychosocial health, culture, religion, migrants, COVID-19, refugees

## A short background to the area of research

International migration has reshaped demographic and social structures around the world. Migration background is in many contexts a strong predictor of social and economic disadvantage. At the center of a number of UN's global Sustainable Development Goals,^1^ migration and integration entail challenges and opportunities for countries of origin, transit and destination, that grow in complexity as forecasts indicate that the number of people on the move in the world is on the rise. Earlier migration related health research has chiefly focused on ill-health among migrants, in conceptualizations with limited cross-cultural validity and has often focused on post-migration context only. Many findings and theoretical models are limited to this context. Our focus on resilience meets these limitations.

In the current volume we are invited to delve into studies who aim at understanding people in vulnerable situations, being refugees, asylum seekers, undocumented migrants, who have experienced multifaceted repressions and discrimination prior to and during their journey, but also other populations in times of crises. These studies explore the contextual factors as well as internal resources that are available to cope with crisis and how they dynamically shape a person's sense of agency and wellbeing. A definition of resilience needs to capture both the individual qualities and the individual's social ecology for recovery and sustainable wellbeing when under extreme stress (Ungar, 2008, 2012), and also to capture the structural factors, on how the societal institutions impact the individual (Panter-Brick and Eggerman, 2012). An approach to resilience needs to avoid transferring the responsibility from the state to the individual, and missing to emphasize the power, agency, and inequality issues in place (Krüger, 2018; Kaya, 2023). Our Research Topic contributes to the wider research literature on resilience among migrants and refugees, which has covered a range of different topics: social determinants, trauma and psychosocial treatment, cultural approach in psychiatry, personal and social forms of resilience, social support, youth self-esteem, community approach, social entrepreneurship, agency, healthcare policy, trafficking survivors, detention, among other topics (Simich and Andermann, 2014).

## Aims and objectives

This Research Topic addresses how individuals in risk express resilience and coping, and how they are enduring the most dire consequences of recent crises. Additionally, the papers conceptualize resilience on individual and community levels. Attention is paid to context and culture, to understand meaning systems as resources for health and wellbeing.

## Presenting the contributing articles, with the main research question, method, material and result

This Research Topic consists of eight papers covering a range of geographical contexts, theoretical frameworks and methodological approaches. Within this diverse background, they address the core set of questions covered by our Research Topic, resilience and coping as expressed among and in relation to migrants, refugees, or asylum seekers. Their findings improve our understanding of resilience today amidst the dynamic setting of migration in an era of a global crisis.

The external factors of resilience, mainly obstacles, is the focus of Bradby et al. article. Based on interviews with service providers the paper considers how migrants' health care needs are addressed and which factors hinder access at organizational and individual levels. Two main obstacles discussed are denying claims based on citizenship and the lack of means of asserting the human right to health.

Similarly, social determinants, in terms of material and interpersonal variables, during pre- and post-migration are found to be central factors for the outcome of resilience, shown in the interview study among Iraqi migrants to Sweden, by Çetrez et al.. The authors point out the lack of informational and emotional support, combined with a lack of support from authorities and a schematic view of the acculturation process, as unmet basic needs for resilience, especially so for women. At the same time, the perception of illness and the help-seeking behavior among Iraqis' points to a strong level of coping, meaning-making, and goals in life, important for the understanding of resilience.

Resilience, both as a personality trait and a coping mechanism is the focus of an Italian study, by Murphy et al.. They investigate the contextual and cross-cultural dimension of resilience, by examining the psychometric properties of the Italian adaptation of the BRCS, for migrant and non-migrant participants. Interestingly, no differences of resilience are found between the migrant and non-migrant populations, but, instead economic, living conditions, gender, and age. Specific for the migration population is the sense of belonging, which correlated with resilience.

Srivastava et al. examine the stress placed upon an already vulnerable group by the COVID-19 pandemic, the internal migrant workers in India. The interviews reveal the many dimensions of deprivation and isolation they have experienced, as well as means of coping: Focusing on problem solving strategies, receiving support from the social environment and maintaining positive thoughts amidst challenges.

While Srivastava et al.'s interviews include male migrants only, this study is complemented by two others which focus on women: Kanal and Rottmann's interviews in Turkey with Syrian Muslim women and Hawkes, Norris, Joyce and Paton's interviews with women of refugee background in Australia. Both studies focus on women's subjective experiences and narratives and also encourage us to reconceptualize refugee agency and resilience. Kanal and Rottmann approach refugee women beyond the dichotomy of either a victim or a defeater of patriarchal culture. Their study identifies faith-based practices, home-making activities and identity building as sources of coping. The study's attention to the stressors within the larger social context and its appreciation of religion, creation or recreation of everyday rituals at home as sources of coping are striking.

In the same vein, Hawkes et al. interviews with women of refugee background and the service providers in Australia aim to contribute to a reconceptualization of resilience that extends beyond a Western hegemonic utilization of the term. The interviews suggest that religion and faith, finding a community, and connections with the host community are factors that contribute to wellbeing. In addition, the study also emphasizes the significance of service providers who function as the first point of contact as well as a continual resource in linking refugees to the host environment.

Understanding meaning-making coping methods is the focus of an Iranian study, by Ahmadi et al.. The results show that religious and spiritual coping methods function with secular coping methods in a parallel worldview system, rather than in a sharp distinction between the systems, which is explained by secularism not being deeply rooted in Iranian's way of thinking.

The final piece in this Research Topic by Henriquez et al. examines the Colombian migrants living in Chile. This study explores identity indicators and thus expands our Research Topic's focus to dynamics of identity as linked to mental health and migration. The results identify that collective self-esteem and ethnic identity correlate positively with all dimensions of psychological wellbeing whereas identity fusion with the country of origin or host country correlates positively with single dimensions of wellbeing.

## In sum, …

As the studies in this Research Topic show, meaning-making, resilience and coping, being individual and collective, as well as social, psychological, and existential in nature, are important abilities that protect the individual against negative effects of stressful events. Investigation of the psychosocial effects of resilience and coping and their contributions to the development of protective strategies in terms of mental and physical health and case management process show the importance of new knowledge. This Research Topic contributes to our understanding of a variety of context and culture based resources; the articles also invite us to think about new ways to conceptualize resilience, to design research and to address ethical and policy-related questions posed by current global crises.

## Author contributions

ÖC: Conceptualization, Project administration, Writing—original draft, Writing—review and editing. SK: Conceptualization, Project administration, Writing—original draft, Writing—review and editing.

## Footnotes

^1^Concerning health, education, gender equality, work, global inequalities, sustainable cities, efforts to combat climate change, promote peaceful and inclusive societies and provide justice for all UN's global Sustainable Development Goals 3, 4, 5, 8, 10, 11, 13, and 16. See IOM (2018).
